# Poor housing in childhood and high rates of stomach cancer in England and Wales.

**DOI:** 10.1038/bjc.1990.129

**Published:** 1990-04

**Authors:** D. J. Barker, D. Coggon, C. Osmond, C. Wickham

**Affiliations:** MRC Environmental Epidemiology Unit, University of Southampton, Southampton General Hospital, UK.

## Abstract

In a search for aetiological processes which might explain the association of stomach cancer with poverty, we have related mortality from the disease in the local authority areas of England and Wales during 1968-78 to indices of living standards derived from the 1971, 1951 and 1931 censuses. We have also analysed recently released data from a national survey of overcrowding carried out in 1936. Geographical differences in stomach cancer were most closely related to occupationally derived indices of socio-economic structure from the 1971 census, and to measures of domestic crowding from the 1931 census and 1936 survey. Unlike other indices of poor living standards, levels of past domestic crowding in north-west Wales were consistent with its previously unexplained high death rates from stomach cancer. We conclude that overcrowding in the home during childhood may be a major determinant of stomach cancer, and might act by promoting the transmission of causative organisms.


Br. J. Cancer (1990), 61, 575-578 © Macmillan Press Ltd., 1990

### Poor housing in childhood and high rates of stomach cancer in England and Wales

D.J.P. Barker, D. Coggon, C. Osmond & C. Wickham

*MRC Environmental Epidemiology Unit, University of Southampton, Southampton General Hospital, Southampton SO9 4XY, UK.*

**Summary** In a search for aetiological processes which might explain the association of stomach cancer with poverty, we have related mortality from the disease in the local authority areas of England and Wales during 1968-78 to indices of living standards derived from the 1971, 1951 and 1931 censuses. We have also analysed recently released data from a national survey of overcrowding carried out in 1936. Geographical differences in stomach cancer were most closely related to occupationally derived indices of socio-economic structure from the 1971 census, and to measures of domestic crowding from the 1931 census and 1936 survey. Unlike other indices of poor living standards, levels of past domestic crowding in north-west Wales were consistent with its previously unexplained high death rates from stomach cancer. We conclude that overcrowding in the home during childhood may be a major determinant of stomach cancer, and might act by promoting the transmission of causative organisms.

In England and Wales mortality from stomach cancer in social class V is two to three times that in social class I (Office of Population Censuses and Surveys, 1982, 1986). Similar associations with poverty have been observed in other western countries (see Howson *et al.*, 1986), but the explanation is uncertain. Differences in diet could be one reason, since various foods and nutrients have been linked with the disease. The strongest evidence is for a protective effect of fresh fruit and salad vegetables and for a causal role of salt (see Coggon & Acheson, 1984). However, in a recent case-control study the association with low social class was little affected by allowing for the large risks associated with these foods (Coggon *et al.*, 1989). It is therefore worth exploring the possibility that other aspects of poverty are important in the causation of stomach cancer.

One potential source of clues is the marked geographical variation in the incidence of stomach cancer within England and Wales. Mortality in rural north-west Wales and in some northern industrial towns is twice that in much of south-east England (Gardner *et al.*, 1983). Information on the geographical distribution of various socio-economic variables is available from censuses. In an attempt to define more closely the features of poverty which predispose to stomach cancer, we have examined the geographical relation between these variables and mortality from stomach cancer during 1968-78. Because studies of migrants indicate that a major component of stomach cancer risk is determined early in life (see Coggon & Acheson, 1984; Coggon *et al.*, 1990), we have used census data not only from the period in which deaths occurred, but also from 1951 and 1931. We have also analysed recently released data from a government survey of housing carried out in 1936.

### Materials and methods

The Office of Population Censuses and Surveys made available extracts from all death certificates in England and Wales during 1968-78. The information provided included age, sex, cause of death and local authority area of residence. For the period April 1969 to December 1972 place of birth was also recorded, although in less geographical detail. County boroughs (large towns) and London boroughs were distinguished, but the rest of the country was classified only by county.

Indices of housing, income, family size, crowding and education were derived from the 1971, 1951 and 1931 censuses of England and Wales (Office of Population Censuses and Surveys, 1971; General Register Office, 1954-5; Registrar General, 1932-6 *a*). Additional data on crowding came from a survey carried out in 1936 by 1,484 of the 1,536 local authorities in England and Wales (Ministry of Health, 1936). The purpose of the survey was to measure the number of working class households which were overcrowded. It used criteria for domestic overcrowding defined by the 1935 Housing Act. For any dwelling a limit was set to the number of people allowed to sleep there. The limit was determined by the number of rooms, their floor area, and the number and ages of the occupants. The survey was directed at working class accommodation and the criteria by which houses were selected for investigation varied between areas. However, because its aim was to identify sub-standard housing, ascertainment of crowded dwellings is likely to have been near complete. From the survey we derived an overcrowding index for each of the 80 county boroughs and 15 London boroughs, for the aggregates of smaller towns within each of the 59 counties, and for the aggregates of rural districts within each county. (One county, Middlesex, contained no rural districts.) The index was defined as

$$\log_{10} \frac{\text{proportion of local population in crowded dwellings} \times 100}{\text{proportion of national population in crowded dwellings}}$$

The denominator populations came from the Registrar General's 1936 annual report (Registrar General, 1937). A log transformation was used because the untransformed data were skewed. Levels of crowding in the 52 small towns and rural districts for which information was missing were assumed to be equal to those for the remainder of the county aggregate to which each belonged.

Using population data from the 1971 census, we calculated age- and sex-standardised mortality ratios for stomach cancer at ages 35-74 during 1968-78 for the same boroughs and county aggregates. We then related the distribution of mortality to that of socio-economic and overcrowding indices in these 212 areas. For comparison, a similar analysis was carried out for other leading causes of death during 1968-78. We also examined the relation of overcrowding in 1936 to PMRs for stomach cancer during 1969-72 calculated by place of birth, and to cause-specific mortality in infants during 1931-35 (Registrar General, 1932-6 *b*). Because place of birth was recorded in less detail than place of death, it was analysed for 159 rather than 212 areas (80 county boroughs, 15 London boroughs and 59 administrative counties).

Associations between socio-economic indices and mortality were examined by scatter plots and quantified by correlation

Correspondence: D. Coggon.
Received 10 August 1989; and in revised form 27 October 1989.

coefficients. The values of the coefficients reflect not only the strength of associations but also the number of deaths from which they are derived. If a relation exists, the coefficient describing it will tend to have a larger absolute value with increasing numbers of deaths.

### Results

The standardised mortality ratios for stomach cancer in the 212 local authority aggregates during 1968–78 at ages 35–74 ranged from 63 in East Sussex, 65 in Eastbourne and 68 in West Sussex to 156 in Caernarvonshire and Gateshead and 160 in Oldham. Ratios were low in East Anglia and southern England, except in a number of London boroughs. Ratios were high in Wales and in areas of central and northern England including Staffordshire, Lancashire, Yorkshire, Durham and Tyneside.

The relation between stomach cancer mortality and indices from the 1971 census is summarised in Table I. The highest correlations ($r = 0.69$) were with the proportion of economically active or retired persons in socio-economic group 11 (unskilled manual workers) and socio-economic groups 10 (semi-skilled manual workers) and 11 combined. Of the household amenities examined, the strongest associations were the proportions of households lacking a car ($r = 0.55$) and without exclusive use of an inside water closet ($r = 0.52$). Domestic crowding, measured by the proportion of households with more than one person per room, was also related to stomach cancer ($r = 0.46$). The correlation with the proportion of persons in socio-economic groups 10 and 11 is illustrated in Figure 1. Places in north-west Wales depart from the overall trend in that their stomach cancer mortality is higher than would be predicted from their socio-economic structure.

Table II shows the correlation coefficients between stomach cancer mortality and indices from the 1951 census. The highest correlations were with the proportion of occupied and retired men in social class V ($r = 0.55$) and the proportion of households having more than one person per room ($r = 0.53$). There were lower correlations with the proportion of men in social classes IV and V combined, and the proportion of men leaving school at a younger age. Correlations with lack of fixed baths, cooking stoves, kitchens sinks, piped water supplies and water closets were small or negative.

Table III shows correlation coefficients for indices from the 1931 census, which did not record social class or information about household amenities. The highest correlation was with the proportion of people in private families with more than one person per room ($r = 0.60$). Correlations with the proportion of families comprising six or more people, and the proportion of families living in houses with only one or two rooms were lower.

We examined the correlation coefficients between the overcrowding index and the 25 leading causes of death at ages 35–74 during 1968–78. Leading causes of death were those for which more than 10,000 deaths occurred in each sex, or in the sex usually affected, during 1968–78. The coefficient for stomach cancer ($r = 0.64$) was higher than that for any other cause of death and equalled that for mortality from all causes combined. (The latter was based on a much larger number of deaths which of itself would tend to give a higher correlation coefficient.) Coefficients for mortality from bronchitis and chronic rheumatic heart disease ranked next below that for stomach cancer. The coefficients for stomach cancer

**Figure 1** Mortality from stomach cancer in men and women aged 35–74 during 1968–78 and proportion of economically active or retired persons in socio-economic groups 10 and 11 at the 1971 census in 212 areas. ●, North-west Wales.

**Table I** Correlations between stomach cancer mortality (standardised mortality ratios both sexes, ages 35–74 1968–78) and indices derived from the 1971 census in the 212 areas of England and Wales

| *Index* | *Correlation coefficient* |
|---|---|
| Proportion of economically active or retired persons in socio-economic group 11 (unskilled manual workers, not self-employed) | 0.69 |
| Proportion of economically active or retired persons in socio-economic groups 10 and 11 (semi-skilled and unskilled manual workers, not self-employed) | 0.69 |
| Proportion of households without a car | 0.55 |
| Proportion of households without exclusive use of inside WC | 0.52 |
| Proportion of households with more than one person per room | 0.46 |
| Proportion of employed persons without ordinary national or school certificate or 'A' level | 0.41 |
| Proportion of households without exclusive use of a bath | 0.33 |
| Proportion of households with more than 0.75 persons per room | 0.26 |
| Proportion of households with more than 1.5 persons per room | 0.21 |
| Proportion of households without exclusive use of hot water | 0.20 |

**Table II** Correlations between stomach cancer mortality (standardised mortality ratios both sexes, ages 35–74, 1968–78) and indices derived from the 1951 census in the 212 areas of England and Wales

| *Index* | *Correlation coefficient* |
|---|---|
| Proportion of employed or retired men in social class V | 0.55 |
| Proportion of households with more than one person per room | 0.53 |
| Proportion of employed men who left school before age 15 | 0.35 |
| Proportion of employed or retired men in social classes IV and V | 0.31 |
| Proportion of 16-year-old boys not in full-time education | 0.28 |
| Proportion of households without exclusive use of fixed bath | 0.22 |
| Proportion of households without exclusive use of a cooking stove | 0.22 |
| Proportion of households without exclusive use of a kitchen sink | 0.01 |
| Proportion of households without exclusive use of a piped water supply | − 0.14 |
| Proportion of households without exclusive use of a water closet | − 0.23 |

**Table III** Correlations between stomach cancer mortality (standardised mortality ratios both sexes ages 35–74, 1968–78) and indices derived from the 1931 census in the 212 areas of England and Wales

| *Index* | *Correlation coefficient* |
|---|---|
| Proportion of people in private families with more than one person per room | 0.60 |
| Proportion of people in private families with more than two persons per room | 0.54 |
| Proportion of people in private families with more than three persons per room | 0.44 |
| Proportion of private families living in four or less rooms | 0.44 |
| Proportion of private families with more than six persons | 0.39 |
| Proportion of private families living in two or less rooms | 0.29 |

were consistently high in each kind of area (Table IV). Figure 2 shows the relation between the overcrowding index and stomach cancer in more detail. In contrast to Figure 1, places in north-west Wales conform to the overall pattern. There was a similar level of correlation when the overcrowding index was related to stomach cancer PMRs by place of birth for deaths during April 1969 to December 1972 ($r$ based on 159 areas = 0.61).

Table V shows the relation of the overcrowding index to infant mortality during 1931–35. As would be expected, overcrowding is highly correlated with post-neonatal mortality and more specifically with post-neonatal mortality from infective diseases.

### Discussion

Our analysis of census data shows the expected geographical correlation between mortality from stomach cancer and measures of socio-economic status. The strongest associations were with two indices from the 1971 census: the proportion of economically active or retired persons in socio-economic group 11, and the proportion in socio-economic groups 10 or 11 ($r = 0.69$ for each). These measures, however, are based on broad occupational groupings, and the correlations do not point to specific aetiological mechanisms. Moreover, differences in the distribution of socio-economic groups 10 and 11 do not account for the high rates of stomach cancer in north-west Wales (Figure 1).

**Table IV** Correlations between causes of death (standardised mortality ratios both sexes, ages 35–74, 1968–78) and overcrowding in 1936

| *Cause of death* | *95 county and London boroughs* | *59 urban aggregates within counties* | *58 rural aggregates within counties* | *All 212 areas* |
|---|---|---|---|---|
| Stomach cancer | 0.59 | 0.64 | 0.70 | 0.64 |
| Bronchitis | 0.53 | 0.70 | 0.61 | 0.60 |
| Rheumatic heart disease | 0.48 | 0.52 | 0.45 | 0.53 |
| All causes | 0.56 | 0.69 | 0.52 | 0.64 |

**Figure 2** Mortality from stomach cancer in men and women aged 35–74 during 1968–78 and overcrowding in 1936 in 212 areas. ●, North-west Wales.

Of all the more specific indices of poverty that we examined, the strongest associations were with measures of domestic crowding, particularly in 1951 and 1931 (Tables II and III). The correlation with domestic crowding was even stronger when based on the index derived from the 1936 local authority survey. The findings of this government survey have been held under a 50 year rule and only recently released. They allow overcrowding to be measured more accurately than from census data because they take into account the size of rooms as well as their number.

In contrast to socio-economic structure, levels of overcrowding in north-west Wales in 1936 were consistent with its current high rates of stomach cancer (Figure 2). The poor housing in Wales before the Second World War, especially in rural areas, was described in a government report on tuberculosis published in 1939 (Ministry of Health, 1939). Among the defects listed were 'small rooms', 'absence of proper larders or pantries' and 'inadequate or entire absence of sanitary facilities'.

Since the Second World War there has been extensive demolition of old houses and new house building. Together with the falling birth rate this has changed the pattern of domestic crowding. That stomach cancer mortality during 1968–78 is more closely related to domestic crowding in the 1930s than in 1951 or 1971 suggests that the association reflects causes of stomach cancer acting during childhood rather than later in life. This would be consistent with studies of migrants (see Coggon & Acheson, 1984; Coggon *et al.*, 1990), and with two recent case–control studies (one carried out in north-west Wales) which have shown a higher risk of disease in people who spent their childhood in areas of high incidence (Coggon *et al.*, 1989; Galpin *et al.*, 1988).

The relation of stomach cancer to past crowding is unlikely to be an artefact of selective migration. Data on mortality by place of birth were only available for 3 years and 9 months, and because appropriate population denominators were not available, it was only possible to calculate proportional mortality ratios. However, despite the smaller number of deaths and the scope for bias because other causes of death may also be related to crowding, the correlation with the overcrowding index was almost as high as for SMRs by place of death.

One possible explanation for the association of stomach cancer with overcrowding is that in the past small houses lacked facilities for food storage, a deficiency described in north Wales (Ministry of Health, 1939), for example. A link between stomach cancer and poor food storage is supported by two case–control studies (Coggon *et al.*, 1989; Risch *et al.*, 1985) and, if real, might explain part of the world-wide decline in the disease over recent decades. In our analysis, however, stomach cancer correlated less strongly with the

**Table V** Correlation of overcrowding in 1936 with infant mortality during 1931–35 in the 212 areas of England and Wales

| *Infant mortality* | *Correlation coefficient* |
|---|---|
| Neonatal | 0.40 |
| Post-neonatal | 0.73 |
| Bronchitis | 0.62 |
| Measles | 0.53 |
| Cerebrospinal fever | 0.48 |
| Whooping cough | 0.40 |
| Diarrhoea | 0.58 |

proportion of families living in small houses than with the proportion in overcrowded dwellings (Table III).

We suggest, therefore, that the association with overcrowding points to an infection transmitted from person to person. The close link between overcrowding and respiratory and enteric infections is illustrated by its association with infant deaths from bronchitis and diarrhoea (Table V). Moreover, bronchitis and rheumatic heart disease, which were the two other major causes of death most closely related to the overcrowding index, both have an infective aetiology in childhood. The relation of stomach cancer to crowding was stronger than for either of these diseases. Among possible organisms which could influence the risk of stomach cancer, *Campylobacter pylori* is a current focus of research. Infection by this organism causes chronic antral gastritis (Dooley & Cohen, 1988). This form of gastritis could, like chronic atrophic gastritis, predispose to stomach cancer, although the link has not yet been documented.

Our findings indicate that housing during childhood may be an important environmental determinant of stomach cancer. This could explain many of the differences in incidence within Britain, including the high rates in north-west Wales. Detailed records from the 1936 overcrowding survey have survived in a number of areas, and we are now examining the risk of stomach cancer in individuals in relation to the structure and size of the houses that they lived in as children.

### References

COGGON, D. & ACHESON, E.D. (1984). The geography of cancer of the stomach. *Br. Med. Bull.*, **40**, 335.

COGGON, D., BARKER, D.J.P., COLE, R.B. & NELSON, M. (1989). Stomach cancer and food storage. *J. Natl Cancer Inst.*, **81**, 1178.

COGGON, D., OSMOND, C., BARKER, D.J.P. (1990). Stomach cancer and migration within England and Wales. *Br. J. Cancer*, **61**, 573.

DOOLEY, C.P. & COHEN, H. (1988). The clinical significance of *Campylobacter pylori*. *Ann. Intern. Med.*, **108**, 70.

GALPIN, O.P., WHITAKER, C.J., WHITAKER, R. & 4 others (1988). Stomach cancer in north west Wales 1982–87. Report to Gwynedd Health Authority. University College of North Wales: Bangor.

GARDNER, M.J., WINTER, P.D., TAYLOR, C.P. & ACHESON, E.D. (1983). *Atlas of Cancer Mortality in England and Wales 1968–78*. Wiley: Chichester.

GENERAL REGISTER OFFICE (1954–5). *Census 1951 England and Wales. County Reports*. HMSO: London.

HOWSON, C.P., HIYAMA, T. & WYNDER, E.L. (1986). The decline in gastric cancer: epidemiology of an unplanned triumph. *Epidemiol. Rev.*, **8**, 1.

MINISTRY OF HEALTH (1936). *Housing Act 1935*. Report on the overcrowding survey in England and Wales 1936. HMSO: London.

MINISTRY OF HEALTH (1939). *Report of the Committee of Inquiry into the Anti-tuberculosis Service in Wales and Monmouthshire*. HMSO: London.

OFFICE OF POPULATION CENSUSES AND SURVEYS (1971). Census. *Small Area Statistics (Ward Library)*. OPCS: Titchfield, Hampshire.

OFFICE OF POPULATION CENSUSES AND SURVEYS (1982). *Cancer Mortality by Occupation and Social Class 1851–1971*. HMSO: London.

OFFICE OF POPULATION CENSUSES AND SURVEYS (1986). *Occupational Mortality Decennial Supplement 1979–80, 1982–83, Great Britain*. HMSO: London.

REGISTRAR GENERAL (1932–36*a*). *Census of England and Wales 1931. County Reports*. HMSO: London.

REGISTRAR GENERAL (1932–36*b*). *Statistical Review of England and Wales 1931, 1932, 1933, 1934, 1935. Part 1: Tables, Medical*. HMSO: London.

REGISTRAR GENERAL (1937). *Statistical Review of England and Wales 1936. Part 1: Tables, Medical*. HMSO: London.

RISCH, H.A., JAIN, M., CHOI, N.W. & 6 others (1985). Dietary factors and the incidence of cancer of the stomach. *Am. J. Epidemiol.*, **122**, 947.


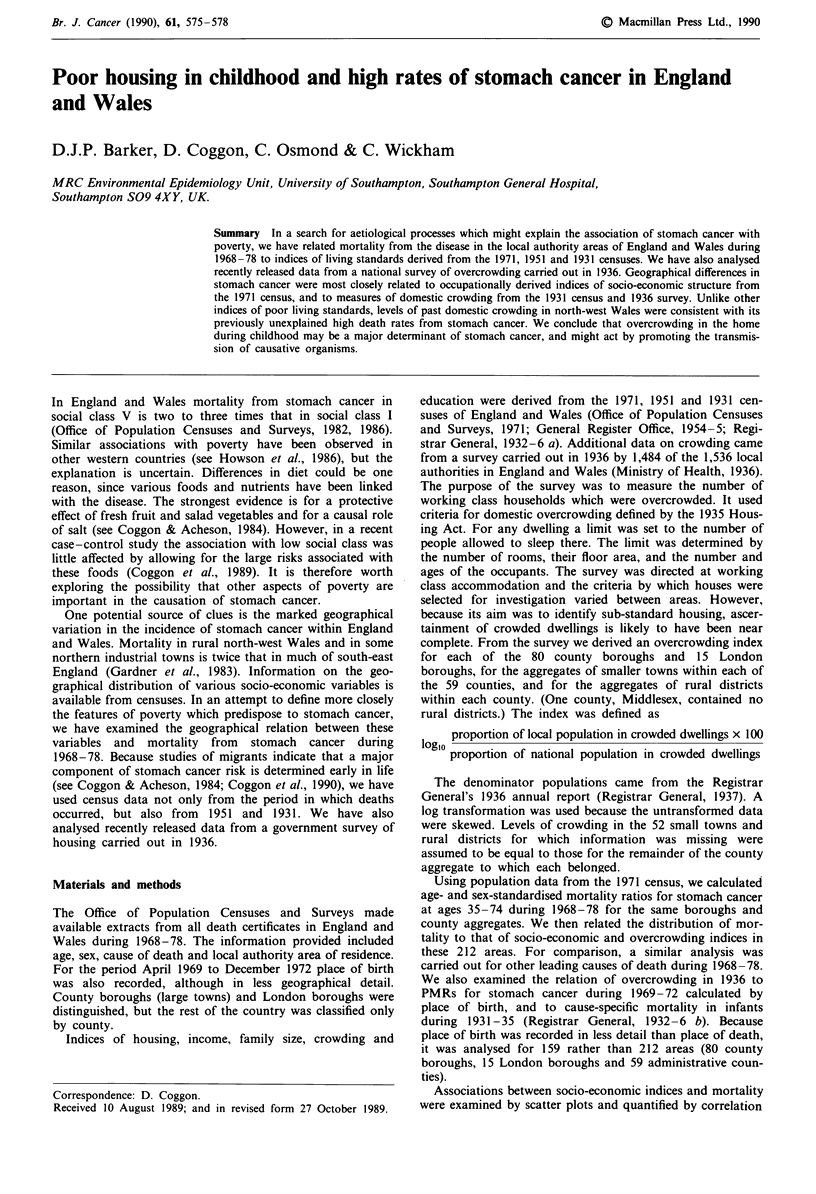

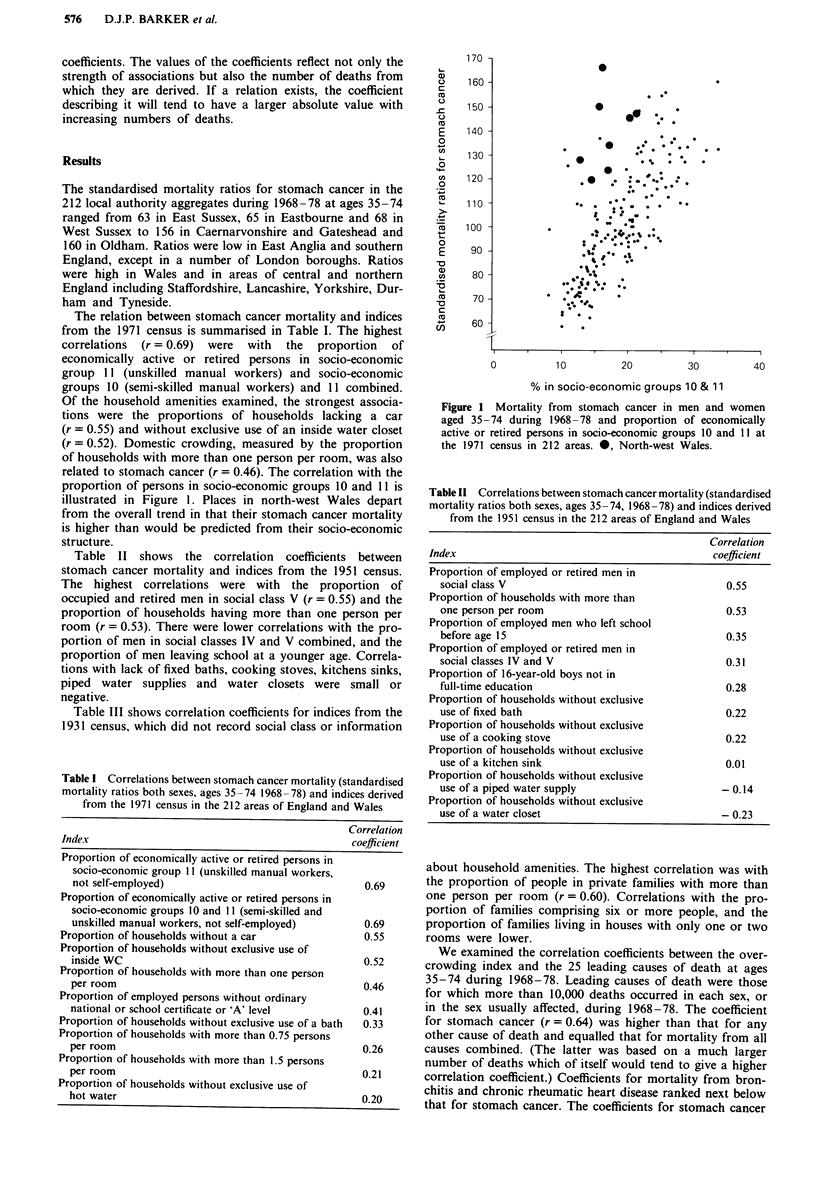

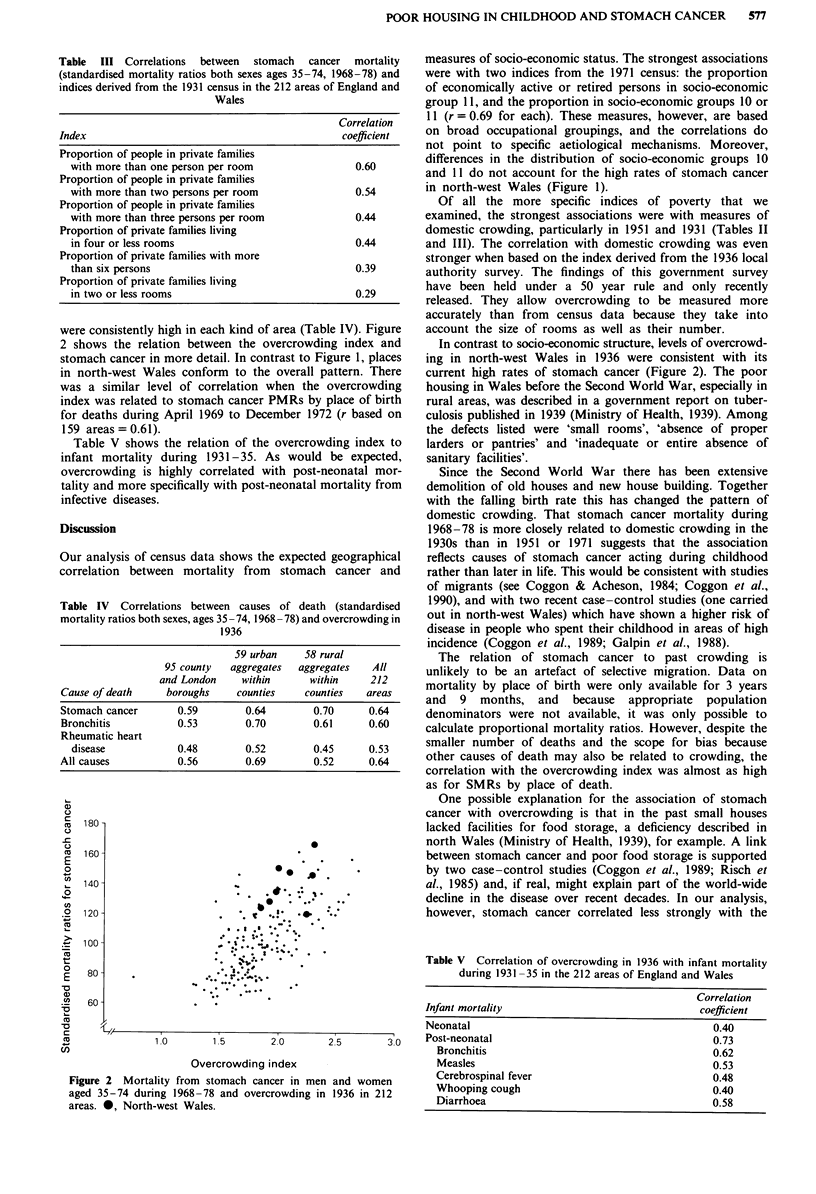

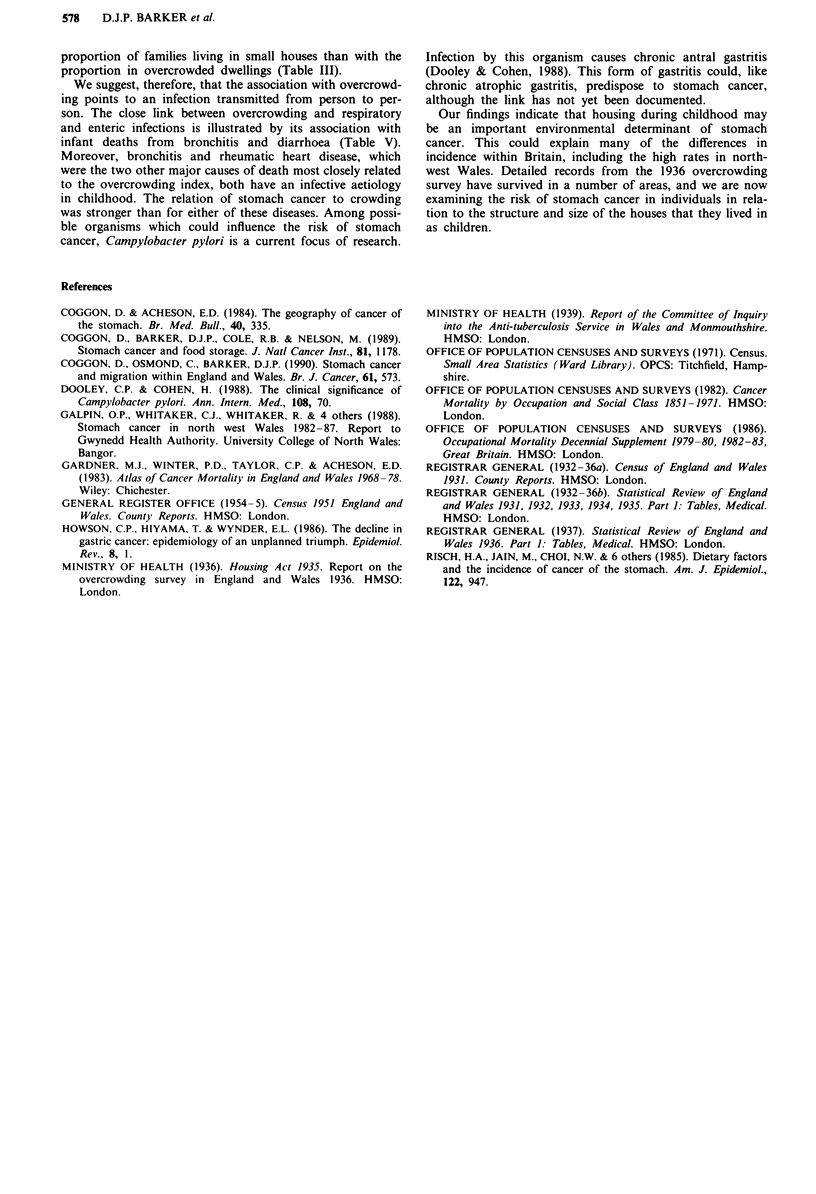


## References

[OCR_00609] Coggon D., Acheson E. D. (1984). The geography of cancer of the stomach.. Br Med Bull.

[OCR_00613] Coggon D., Barker D. J., Cole R. B., Nelson M. (1989). Stomach cancer and food storage.. J Natl Cancer Inst.

[OCR_00616] Coggon D., Osmond C., Barker D. J. (1990). Stomach cancer and migration within England and Wales.. Br J Cancer.

[OCR_00619] Dooley C. P., Cohen H. (1988). The clinical significance of Campylobacter pylori.. Ann Intern Med.

[OCR_00638] Howson C. P., Hiyama T., Wynder E. L. (1986). The decline in gastric cancer: epidemiology of an unplanned triumph.. Epidemiol Rev.

[OCR_00681] Risch H. A., Jain M., Choi N. W., Fodor J. G., Pfeiffer C. J., Howe G. R., Harrison L. W., Craib K. J., Miller A. B. (1985). Dietary factors and the incidence of cancer of the stomach.. Am J Epidemiol.

